# Ultrasonic‐Assisted Synthesis of Highly Defined Silver Nanodimers by Self‐Assembly for Improved Surface‐Enhanced Raman Spectroscopy

**DOI:** 10.1002/chem.201904518

**Published:** 2020-01-21

**Authors:** Junfang Zhang, Soeun Gim, Grigori Paris, Pietro Dallabernardina, Clemens N. Z. Schmitt, Stephan Eickelmann, Felix F. Loeffler

**Affiliations:** ^1^ Department of Biomolecular Systems Max Planck Institute of Colloids and Interfaces Am Mühlenberg 1 14476 Potsdam Germany; ^2^ Department of Biomaterials Max Planck Institute of Colloids and Interfaces Am Mühlenberg 1 14476 Potsdam Germany

**Keywords:** aggregation, nanodimers, self-assembly, surface-enhanced Raman spectroscopy, ultrasonic-assisted synthesis

## Abstract

Considerable research efforts have been devoted to surface‐enhanced Raman spectroscopy (SERS), due to its excellent performance in biosensing and imaging. Here, a novel and facile strategy for the fabrication of well‐defined and uniform nanodimers as SERS substrates is presented. By the assistance of ultrasound, the violent polyol process for particle generation becomes controllable, enabling the self‐assembly of nanostars to nanodimers. Moreover, the aggregation of nanodimers can be easily tuned by post‐ultrasonic treatment, which gives a sensitive substrate for SERS.

## Graphical abstract

**Made of nanostars**: By the assistance of ultrasound, the violent polyol process for silver particle generation becomes controllable, enabling the self‐assembly of nanostars to nanodimers. Moreover, the aggregation of nanodimers can be easily tuned by post‐ultrasonic treatment, which gives a sensitive substrate for SERS.

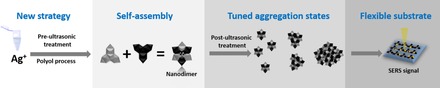

Surface‐enhanced Raman spectroscopy (SERS) has received considerable attention as a powerful tool for bioanalysis.^1^ This technique enhances Raman signals from 4 to 11 orders of magnitude by electromagnetic and chemical enhancement by using metal nanostructures.^2^ In comparison to organic fluorophores, which are standard in sensing and microscope imaging,^3^ SERS shows several advantages: 1) ultrahigh sensitivity, even for single‐molecules,^4^ 2) 10–100 times narrower bandwidth of peaks,^5^ 3) resistance to photobleaching, saturation, and degradation, 4) capability to be driven by near‐infrared light.^6^

Nobel metal nanomaterials, such as gold, silver, or sometimes copper (Au, Ag, Cu), are most commonly used as SERS substrates.^7^ In particular, silver works well for the excitation with wavelengths >400 nm, whereas for gold, excitation wavelengths >600 nm are necessary.2a The plasmonic properties of nanoparticles have been demonstrated to play the key role in SERS.^8^ Thus, the performance of SERS mainly depends on the size, shape, and assembly of metal nanostructures.^9^ Anisotropic nanostructures with peculiar morphology and high roughness exhibit stronger signals.^10^ Yet, it is still challenging to achieve highly defined and flexible SERS substrates. Recently, star‐like nanostructures have been reported to show a high Raman enhancement factor,^11^ but the fabrication of silver nanostars is not well‐reproducible and the aggregation states cannot be flexibly tuned. Thus, the common methods cannot provide well‐defined and controllable anisotropic nanostructures.

In this work, we present a facile way to synthesize a new silver nanostructure with the assistance of ultrasound. We hypothesize that through pre‐ultrasonic treatment, the pre‐produced nanoparticles serve as self‐seeds during the conventional polyol process. This efficient nucleation step leads to the self‐assembly from single nanostars to uniform and stable dimers in the presence of polyvinylpyrrolidone (PVP). The SERS signal produced by these dimers is several times higher than that from single particles. This phenomenon can be explained by the “hot spot” effect,^12^ according to which the localized electric field is strongly enhanced by clusters,^13^ narrow gaps,^14^ and edges^15^ of metal nanomaterials. Moreover, the aggregation of these dimers to larger assemblies can be well controlled by post‐ultrasonic treatment, which helps to fine‐tune this approach for different applications. Finally, this offers new nanostructures for SERS substrate and may also provide a facile strategy for the fabrication of other metal nanomaterials.

A variety of different methods for the synthesis of silver nanoparticles are known, including chemical reduction and physical irradiation.^16^ Chemical reduction is the main choice, because it is more efficient to generate nanoparticles in different shapes and sizes.^17^ One of the most widely used methods in chemical reduction is the so‐called “polyol process”, in which ethylene glycol is used as both reducing reagent and organic solvent. This method has excellent performance for the synthesis of simple nanostructures, such as nanospheres, nanotubes and nanowires.

We chose this polyol process for the synthesis of nanostars. Scanning electron microscopy (SEM) is used to visualize the resulting particle morphology (Figure 1 A). These nanostars are around 20 μm in size, which is rather large and not uniform, therefore, not very suitable as SERS substrates. As a result, this method is less suitable, when it comes to more complex anisotropic structures, such as nanostars. Ethylene glycol in chemical reduction is highly reactive under high temperature (160 °C). The nanostars, which undergo this process, grow rapidly into huge particles with a broad size distribution. It is difficult to synthesize well‐defined and homogeneous nanostars with this polyol process, not to mention the fabrication of nanostars with the ability of flexible aggregation states.

**Figure 1:**
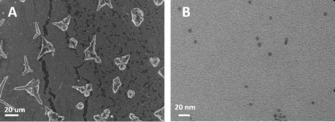
(A) SEM images of silver nanostars obtained by standard polyol process, (B) TEM image of nanospheres obtained from only 30 min sonication without the poly process.

According to La Mer's model,^18^ which describes the formation of nanocrystals, “nucleation” and “growth” are the main governing steps in the synthesis process of nanoparticles. Both are highly dependent on the concentration of available atoms (or atom clusters) in the solution. The process begins with the nucleation step, in which, after an initial burst of nuclei generation, the concentration of available atoms decreases rapidly. Beneath a certain concentration, the nucleation stops and is succeeded by the much slower growth step, which is the rate‐determining step of the process. Hence, nucleation and growth are separated in time. During the growth step, diffusion limits the synthesis process and also the particle size. Therefore, if we can slow down the fast nucleation step by using milder conditions instead of rapid chemical reactions, we may further separate the nucleation and growth steps, to improve the control over the synthesis.

For this reason, it is necessary to find a mild pre‐treatment, which can slow down the synthesis of nanoparticles and make the violent polyol process controllable. Ultrasound is a promising option. We therefore treated the initial silver solution with ultrasound. As shown in Figure 1 B, only small nanospheres of below 5 nm can be found by transmission electron microscopy (TEM), which means that ultrasound can facilitate an efficient nucleation process, but cannot provide enough energy for the nanoparticles to grow further. Therefore, we combined the typical polyol process with ultrasound as a new strategy for the fabrication of well‐defined nanostars with flexible assembly (Scheme 1).

**Scheme 1:**
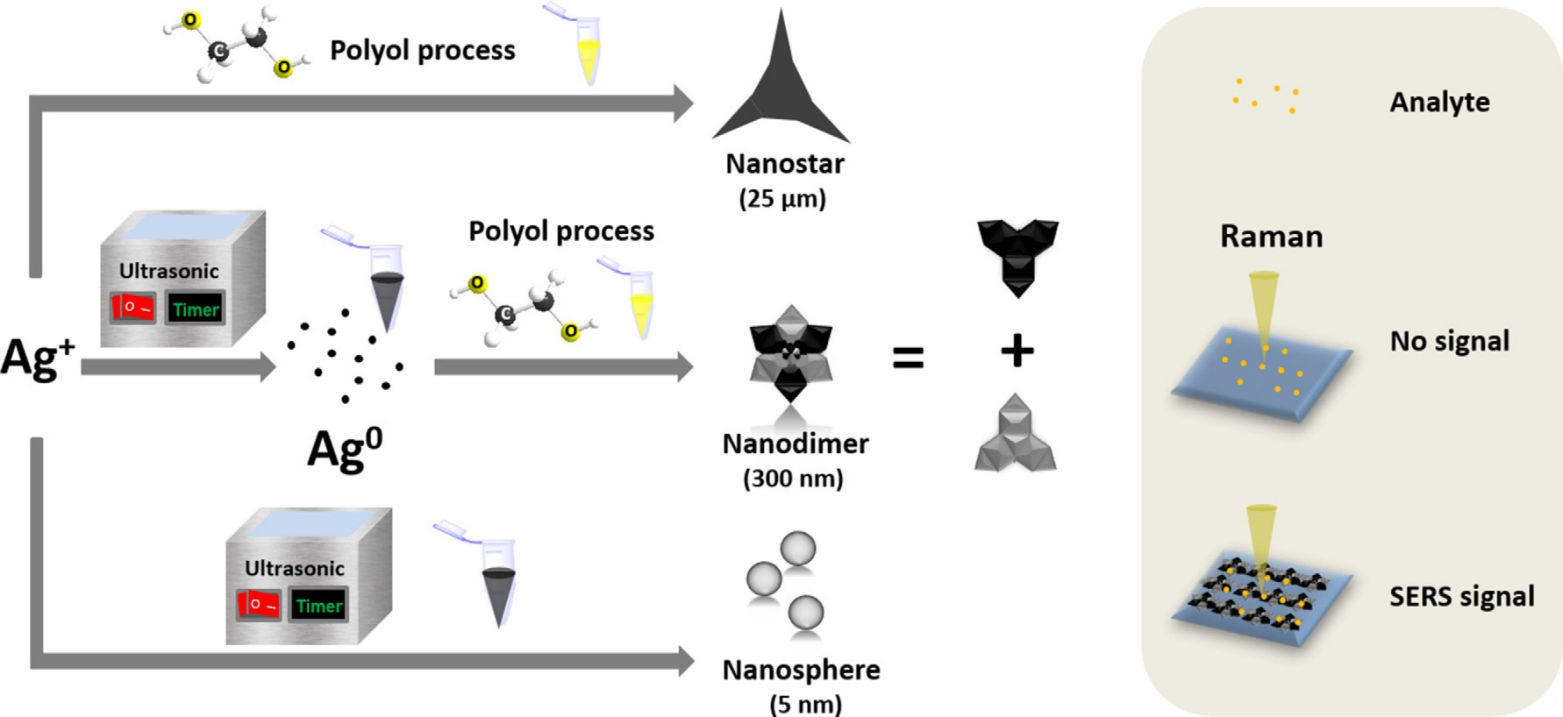
Fabrication of different nanostructures and their application for surface‐enhanced Raman spectroscopy (SERS). The nanostars, obtained from the polyol process, are around 20 μm in size. When the silver salt solution is treated only with ultrasound, tiny nanospheres around 5 nm are formed. In contrast, new nanostructures, which are dimers of nanostars, can be obtained when the initial silver solution is pre‐treated with ultrasound for 30 min before proceeding with a high temperature (160 °C) to start the typical polyol reaction. This nanodimer structure is designed as a tunable substrate for SERS.

First, the solution of silver salt in ethylene glycol is pre‐treated with ultrasound for 30 min, before proceeding with a high temperature (160 °C) to start the conventional polyol reaction. As a result, new nanostructures, which are nanostars that self‐assemble to dimers, can be observed by SEM (Figure 2 A,B and Figure S1 in the Supporting Information for further purification steps). The particles show a well‐defined shape and uniform size with a low polydispersity index (PdI) of 0.182 (Figure 2 E). The nanodimers have an average of 319 nm in size, 100 times smaller than the nanostars, obtained from the conventional polyol process. This also explains the reason for self‐assembly: smaller nanostars have a comparatively larger surface area, so that the minimization of interfacial tension can drive them to form more stable dimers.

**Figure 2:**
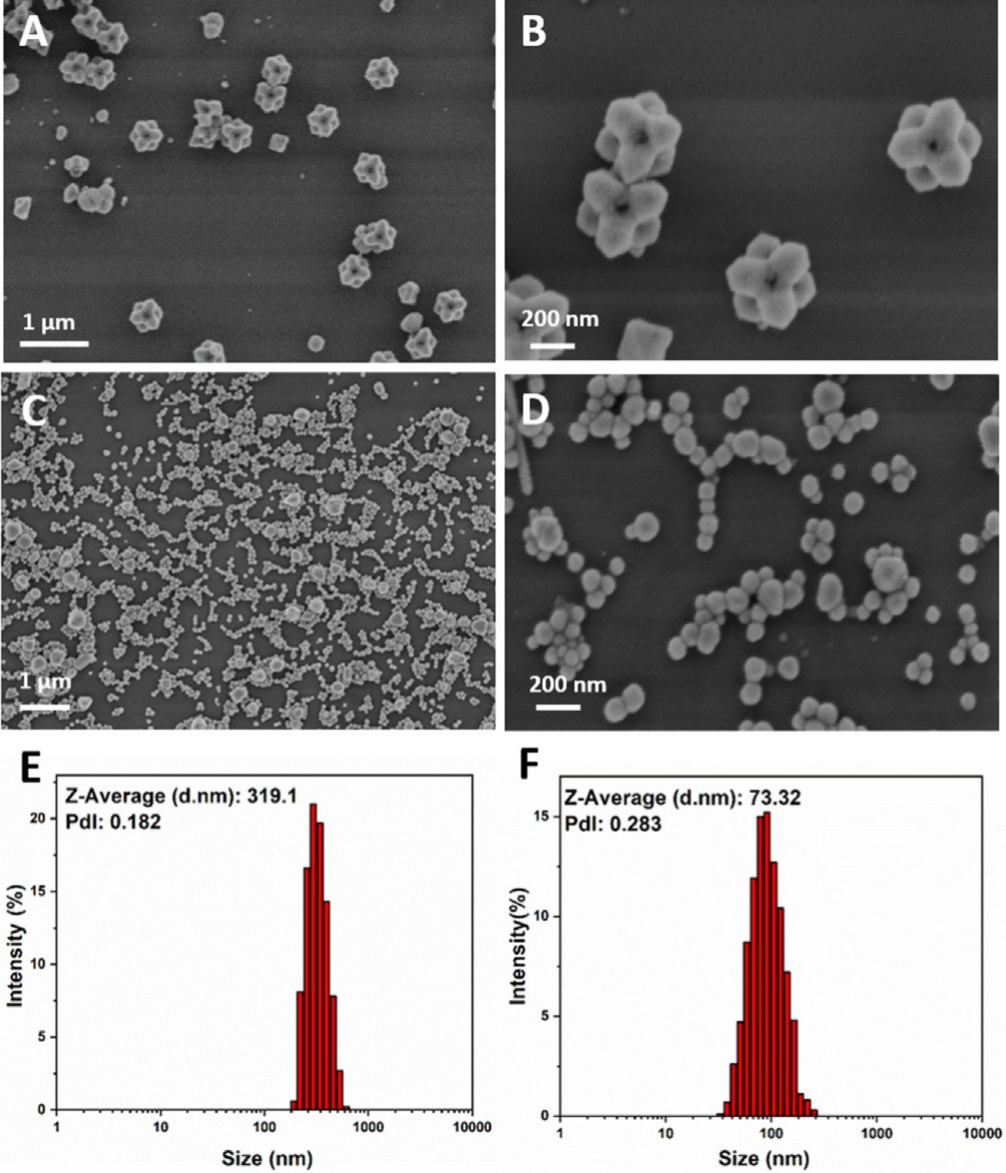
(A),(B) SEM images of silver nanodimers obtained by polyol process with pre‐sonication for 30 min, (Yield: 80 %) (C),(D) nanospheres obtained by polyol process with pre‐sonication for 60 min. (E) Size distributions of silver nanodimers by dynamic light scattering (DLS). (F) Size distributions of silver nanospheres.

The classical Deryaguin–Landau–Verwey–Overbeek (DLVO) theory, a dispersion‐stabilizing theory for colloids,^19^ can be used to further explain the mechanism of self‐assembly. The stability of colloids is based on the equilibrium between the van der Waals attraction and the electrostatic repulsion due to the so‐called “double layer” structure, naturally occurring on suspended particles [Eq. (1)]:^20^

(1) $$V\left(H\right)=V_{R}\left(H\right)+V_{A}\left(H\right)$$

in which *V*, *V_R_*, *V_A_* are the total, (repulsive) electrostatic, and (attractive) van der Waals interaction energies, respectively, and *H* is the separation distance between two particles. At larger distances, long‐range van der Waals attraction is the dominant interaction between particles. However, when they get close to each other, a repulsive barrier occurs mainly because of the short‐range electrostatic repulsion.^21^ If this repulsive barrier is about one order of magnitude smaller than the thermal energy k_B_ *T*, Brownian motion can overcome this barrier.^22^ Here, k_B_ is the Boltzmann constant and *T* is the temperature. As a result, dimerization and even higher degrees of agglomeration can occur in the system.^23^ For ideal interactions, the electrostatic energy (*V_R_*) is expressed by Equation (2):^24^

(2) $$V_{R}\left(H\right)=2\pi \epsilon_{0}\epsilon r_{p}\psi_{p}^{2}ln\left(1+e^{-\kappa H}\right)$$

in which *ϵ_0_* is the dielectric permittivity of vacuum, *ϵ* is the dielectric constant of the solvent, *r_p_* is the average radius of particles, *ψ_p_* is the surface potential of the particles, and *κ* is the inverse Debye length. According to Equation 2, considering that nanoparticles obtained by our ultrasonic‐assisted polyol strategy are much smaller than the particles from the typical polyol process, the repulsive barrier decreases. Therefore, dimerization is more likely to occur in this system.

Moreover, according to the DLVO theory, nanoparticles, especially around 100 nm,^25^ have an optimum force‐to‐mass ratio, leading to efficient dispersion in the solvent, due to Brownian motion. Therefore, also the growth step, which is driven by diffusion, is more defined. This results in more uniform nanodimers with our ultrasonic‐assisted polyol process. In contrast, the larger nanostars, obtained by the typical polyol process, are governed by normal gravity, leading to inefficient growth.

Another interesting result is that, when the solution is treated by ultrasound for a longer time (e.g., 1 h) and then heated to 160 °C to start the conventional polyol process, nanospheres of around 70 nm are generated (Figure 1 C,D). All following measurements are based on these larger nanospheres. During the nucleation step, the monodispersity of atom clusters directly influences the quality and quantity of nuclei. For these longer sonication times, more nuclei are generated, which consumes more available atoms in solution. In this case, the nanocrystals cannot grow further, due to the low atom concentration, which can only support the formation of small nanospheres instead of larger nanodimers. Therefore, ultrasound may also interfere with the growth step, if it is applied for too long.

In addition, we performed X‐ray diffraction (XRD) measurements to analyze the crystallinity of the silver nanoparticles. The peaks recorded from 30° to 80° (Figure 3 A) are assigned to the diffractions from (111), (200), (220), and (311) planes, respectively. Compared with the standard card (JCPDS, file No. 04‐0783), these patterns can be indexed to face centered cubic (FCC) silver. No other peaks are found in the XRD pattern, which indicates high purity of the nanoparticles. This is the advantage of the self‐seeding process. The silver nuclei generated in the first stage serve as seeds for the growth step. Thus, we do not need to introduce exotic seeds, such as platinum nanoparticles,^26^ into the system. This self‐seeding process provides the guarantee for the high purity of nanoparticles.^27^ Figure S2 (Supporting Information) shows the TEM images of the nanoparticles obtained after 10 min at 160 °C. Most of the seeds grow into thermodynamically stable twinned particles with multiply twinned decahedra (MTP) as the dominant shape. The difference between the ultrasonic‐assisted and the normal polyol process is that, without ultrasound, the nuclei are quickly formed within the first minute,^28^ which makes the nucleation step uncontrollable. UV/Vis spectra also provides evidence for this. All three nanoparticle types show an absorption around 400 nm (Figure 3 B), which gives the silver nanoparticle solution during and after reaction a yellow color. We can observe this color immediately, when heating the solution to 160 °C (Figure S3, Supporting Information). However, the XRD measurements for the different nanoparticles show different intensity ratios between the (111) and, for example, (200) peak. This indicates that {100} facets are preferred by nanospheres, but not by nanodimers. The nanoparticles have different directions for growth and subsequently become different shapes.

**Figure 3:**
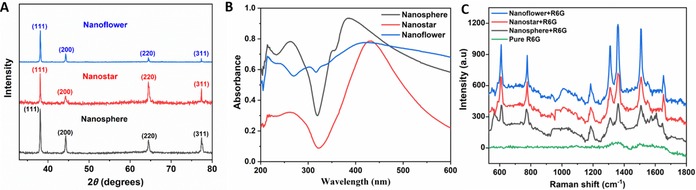
(A) XRD patterns of silver nanoparticles. All three patterns can be indexed to face centered cubic (FCC) silver. However, the intensity ratios between (111), (200), (220), and (311) peaks differ for different nanoparticles. It indicates that they have different preferred facets and prefer in different growth directions. (B) UV/Vis absorption spectra of silver nanoparticles. Nanodimers show a broad absorption. All nanoparticles show absorption around 400 nm, which gives the silver nanoparticles solution during and after reaction a yellow color. (C) SERS spectra of silver nanoparticles with 1×10^−6^ m rhodamine 6G (R6G) in aqueous solution as a Raman probe molecule. Nanodimers show the strongest enhancement of the Raman signal.

Next, we analyzed the performance of the nanodimers in SERS by using rhodamine 6G (R6G) as a model molecule.^29^ Figure 3 C shows the Raman spectra of 1×10^−6^ m R6G on different substrates. When the spectrum of R6G is measured on a bare Si wafer, almost no signal can be observed, due to the low concentration. Weak signals can be found for the nanospheres, mixed with R6G, indicating the enhancing effect. Nanostars are superior to nanospheres, because nanostars have a larger surface roughness and the tips of the star structure lead to stronger localized electric fields (“hot spot” effect). However, the enhancement of the Raman signal is still limited, because of the low quality (size and uniformity) of nanostars.

Finally, our new nanodimer structures exhibit significantly enhanced signals in comparison to the other two nanostructures. Thus, this new structure can be used as a powerful SERS substrate. An excellent SERS substrate should not only enhance the Raman signal sensitively, but also be flexible for a wide range of applications: besides shape and size, the aggregation state of nanoparticles also has a significant influence on the intensity of a SERS signal.4a, 30 As show in Figure 4 A, we treated the nanodimer samples with a post‐synthesis ultrasonic step for different durations (2 or 5 h). Every hour, the distilled deionized H_2_O (dd H_2_O) was exchanged by centrifugation (470 rcf for 20 min) and then redispersed in fresh dd H_2_O. Figure 4 B–D show the SEM images of the nanodimer samples obtained by the above treatment. It is worth noting that these samples exhibit quite different aggregation states. The longer the washing time, the larger the particles become (Figures S4 and S5, Supporting Information) and the more aggregation occurs. In Figure S6 (Supporting Information), the TEM image shows the PVP coating on the nanodimers, which can be observed as the lower contrast areas on the edges of the structures. On the one hand, PVP selectively adsorbs to certain facets to define the oriented growth of nanoparticles. On the other hand, the PVP coating also prevents aggregation of nanoparticles. Considering that the reaction solution is removed, the growth step is stopped before the post‐ultrasonic treatment. The energy provided by ultrasound can remove PVP from the surface of the nanoparticles. As a result, longer ultrasound treatment removes more PVP and causes more aggregation of nanoparticles. The results from energy‐dispersive X‐ray (EDX) spectra (Figure 4 E–G) corroborate this point: the carbon and oxygen content (Table S1, Supporting Information) decreased over time, which gives evidence for the loss of PVP. The different aggregation states of nanodimer particles have different SERS performance (Figure S7, Supporting Information).

**Figure 4:**
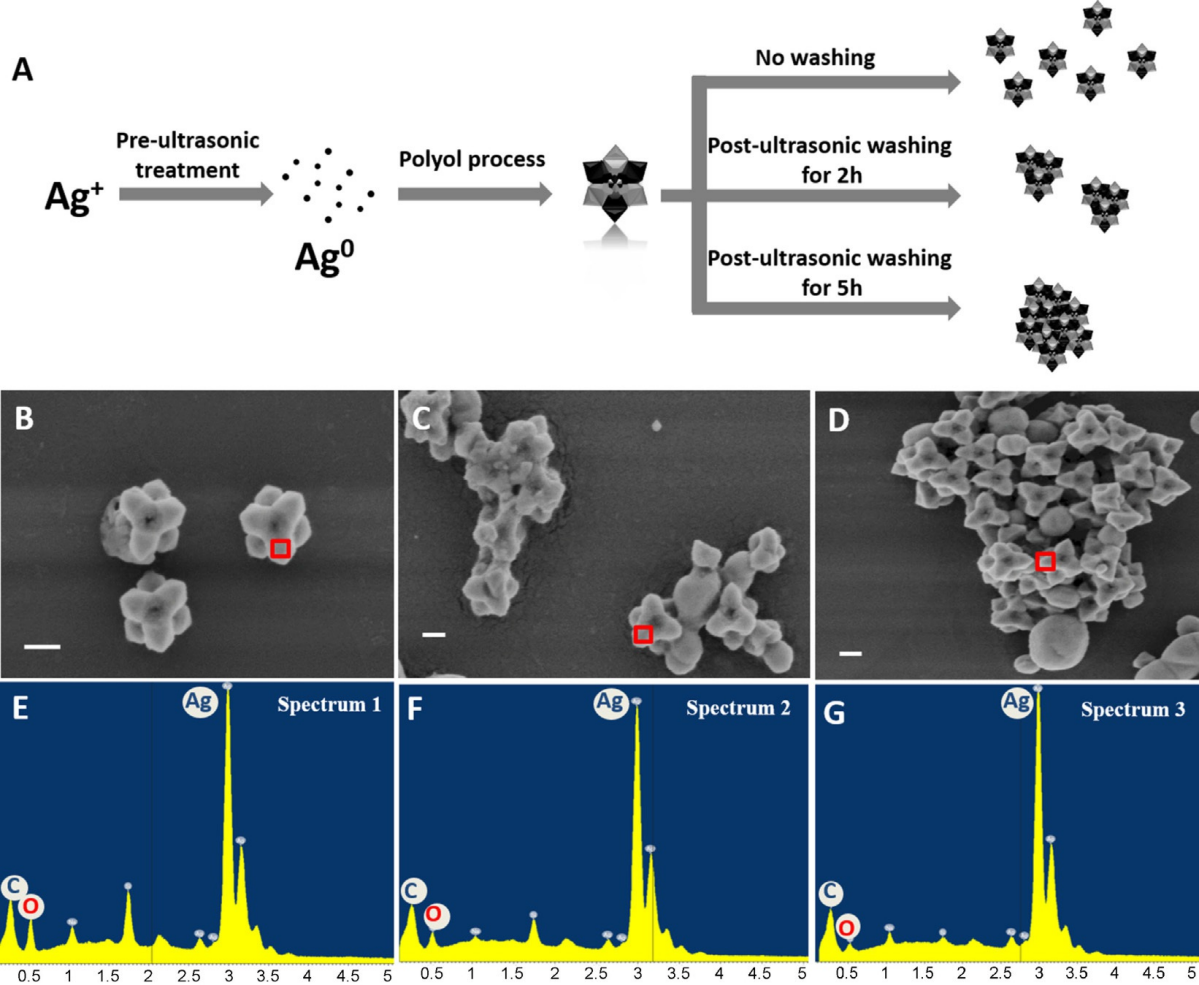
(A) Synthesis of nanodimers with subsequent tuning of the aggregation state via ultrasonic washing. SEM images and energy‐dispersive X‐ray (EDX) spectra of silver nanodimers treated with ultrasound for different times, (B, E) 0 h, (C, F) 2 h, (D, G) 5 h. Longer ultrasonic treatments reduce the oxygen signal (i.e., removing the polymer) and increase the aggregation of nanoparticles (scale bars 200 nm).

In summary, highly sensitive and tunable SERS substrates are important but difficult to fabricate. If we rely on the polyol process alone, it is difficult to obtain well‐defined nanostar structures. Similarly, only with ultrasonic treatment, the energy is just sufficient to create small nanospheres. However, when the polyol process is preceded by a short ultrasonic treatment, this strong chemical reaction becomes more controllable and uniform nanodimers are obtained by the self‐assembly of well‐defined nanostar structures. Moreover, post‐ultrasonic treatment can help to change the aggregation state of these nanodimers, which makes this substrate even more powerful for various applications. This work not only provides a facile way to synthesize novel nanostructures for SERS, but also gives new insights into the fabrication of nanoparticles.

## Experimental Section

**Synthesis of nanoparticles**: The nanostars were prepared by typical polyol process. Silver nitrate (99.999 %) and polyvinylpyrrolidone (PVP, av. *M*w ≈1 300 000) were acquired from Sigma Aldrich. Anhydrous ethylene glycol (EG, 99.8 %) was acquired from Arcos (please note: EG from Acros gave reproducible results with different EG batches; EG from Sigma Aldrich, purity 99.8 %, yielded non‐reproducible results with different EG batches). In brief, 5 mL anhydrous ethylene glycol was heated to 160 °C for 1 h by using an oil bath. As‐prepared Ag seeds (200 μL; 1.6 mg mL^−1^ in ethylene glycol) were introduced. After 2 min, 3 mL of a 0.085 m AgNO_3_ solution and 3 mL of a 0.128 m PVP solution in anhydrous ethylene glycol were slowly added under vigorous stirring. The reaction was kept for 1 h under reflux. Then, the solution was diluted in acetone, followed by a centrifugation step at 470 rcf for 30 min. The precipitates were collected and washed twice with acetone and dd H_2_O. The nanodimers are prepared with the assistance of ultrasound, which is from Bandelin electronic GmbH & Co. KG (RK100 H) with 35 KHz. Before the polyol reaction, 4 mL of 0.085 m AgNO_3_ solution is sonicated for 30 min. 1 mL of this AgNO_3_ solution is used for the seed generation. Afterwards, it is added into heated ethylene glycol together with the PVP solution. The following operations are the same as polyol process.

**Characterization**: Samples were prepared on carbon‐coated copper grids on a Zeiss EM 912Ω instrument at 120 kV for transmission electron microscopy (TEM). Scanning electron microscopy (SEM) images were obtained on glass by a Gemini SEM, LEO 1550 system with cold field emission gun operation at 3 kV. Absorption spectra were collected with a SHIMADZU UV‐vis spectrophotometer (UV‐2600). For XRD measurements, a Bruker D8 Advanced X‐ray diffractometer with Cu_Kα_ radiation was employed. Surface‐enhanced Raman spectroscopy (SERS) was performed with a confocal Raman microscope (CRM200, WITec). A diode‐pumped near IR laser (785 nm, Toptica Photonics AG) was focused on the sample through a 100× objective lens. The laser power on sample was set to 3 mW. The spectra were acquired by using an air‐cooled CCD (DU401A‐DR‐DD, Andor) behind a 300 g mm^−1^ grating spectrograph (Acton, Princeton Instruments Inc.) with a spectral resolution of 6 cm^−1^. 20 μL of 1 mg L^−1^ aqueous suspension of the silver nanoparticles was mixed with 20 μL of 10^−6^ m R6G, and sonicated for 5 min to reach the adsorption equilibrium. Then, 20 μL of the mixture were deposited onto a Si wafer and dried in air at room temperature for SERS detection.

## Conflict of interest

The authors declare no conflict of interest.

## Supporting information

As a service to our authors and readers, this journal provides supporting information supplied by the authors. Such materials are peer reviewed and may be re‐organized for online delivery, but are not copy‐edited or typeset. Technical support issues arising from supporting information (other than missing files) should be addressed to the authors.

Supplementary
