# Supplementary material for: Ultrasonic‐Assisted Synthesis of Highly Defined Silver Nanodimers by Self‐Assembly for Improved Surface‐Enhanced Raman Spectroscopy
Source: Chemistry. 2020 Jan 21;26(6):1243–8. doi: 10.1002/chem.201904518 (PMC7027530; doi:10.1002/chem.201904518)
Supplement: Supplementary file 1 — Supplementary [file CHEM-26-1243-s001.pdf]

CHEMISTRY
A European Journal

Supporting Information

Ultrasonic-Assisted Synthesis of Highly Defined Silver Nanodimers by Self-Assembly for Improved Surface-Enhanced Raman Spectroscopy

Junfang Zhang,[a] Soeun Gim,[a] Grigori Paris,[a] Pietro Dallabernardina,[a] Clemens N. Z. Schmitt,[b] Stephan Eickelmann,[a] and Felix F. Loeffler*[a]

chem_201904518_sm_miscellaneous_information.pdf

Supplementary Information

Ultrasonic-assisted synthesis of highly defined silver nanodimers by self-assembly for improved surface-enhanced Raman spectroscopy

Junfang Zhang, [a] Soeun Gim, [a] Grigori Paris, [a] Pietro Dallabernardina, [a] Clemens N.Z. Schmitt, [b] Stephan Eickelmann [a] and Felix F. Loeffler[a]*

[a] Department of Biomolecular Systems, Max Planck Institute of Colloids and Interfaces, Am Muehlenberg 1, 14476 Potsdam, Germany.

[b] Department of Biomaterials, Max Planck Institute of Colloids and Interfaces, Am Muehlenberg 1, 14476 Potsdam, Germany.

Fig. S1 S2

Fig. S2 S3

Fig. S3 S4

Fig. S4 S5

Fig. S5 S6

Fig. S6 S7

Fig. S7 S8

Table. S1 S9

Supplemental experiment results

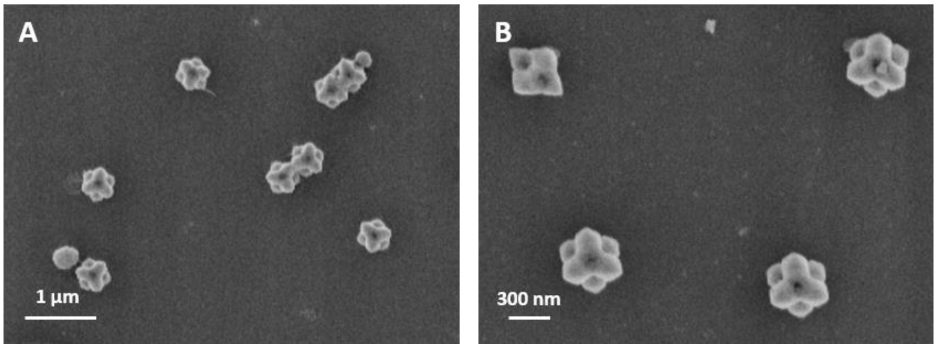

Fig. S1 Scanning electron microscopy (SEM) images of silver nanodimers, washed in a mixture of double-distilled water and acetone (3:2) 5 times at 380 rcf for 15 min.

Centrifugation is a straightforward and useful way for purification. However, the result of purification varies for different nanostructures and nanomaterials. We have tried different solvents (double-distilled water, acetone, ethanol, and their mixtures) at different relative centrifugal forces (75 rcf, 117 rcf, 169 rcf, 264 rcf, 380 rcf, 470 rcf, 568 rcf, 734 rcf, 1057 rcf) for different times (5 min, 10 min, 15 min, 20 min, 25 min, 30 min). The washing step was repeated 2 times, 3 times, 4 times, and even 5 times. As shown in Fig. S1, the nanodimers, washed in the mixture of double-distilled water and acetone (3:2) at 380 rcf for 15 min and 5 times looks much purer than before. However, since the nanoparticles are washed many times, we lose quite a lot of material and slight aggregation appears. Thus, centrifugation may not be the most efficient way for purification of the nanodimers.

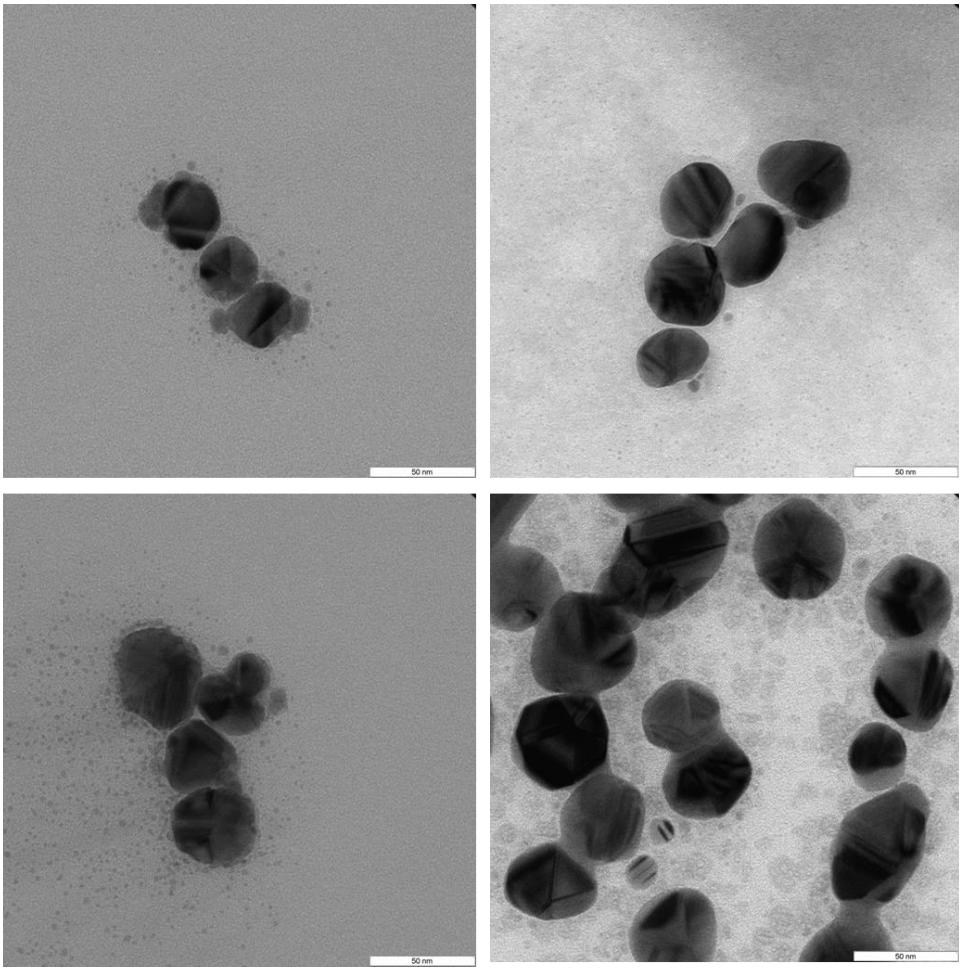

Fig. S2 Typical TEM image of a sample that was prepared from a reaction solution prior to the appearance of silver nanodimers.

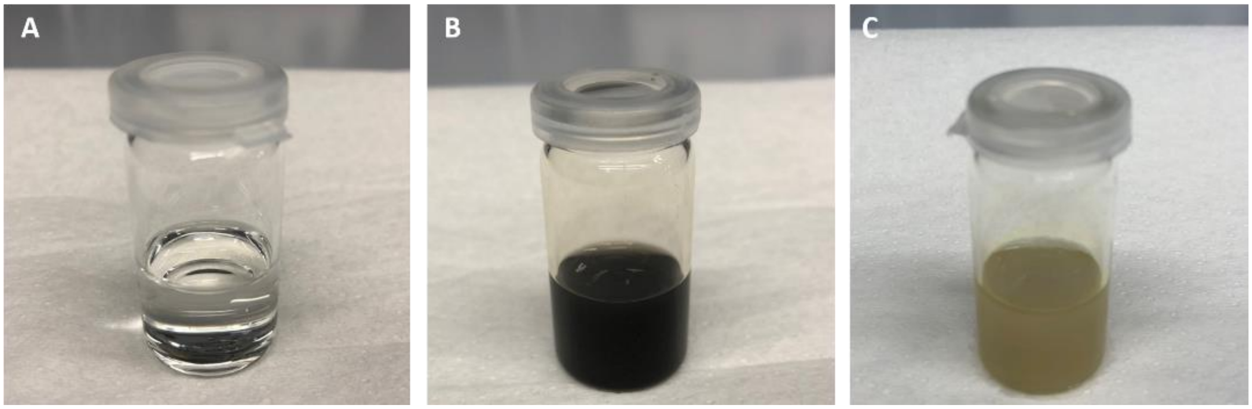

Fig. S3 (A) Solution before all the treatment, (B) solution after pre-ultrasound treatment, (C) solution after polyol process.

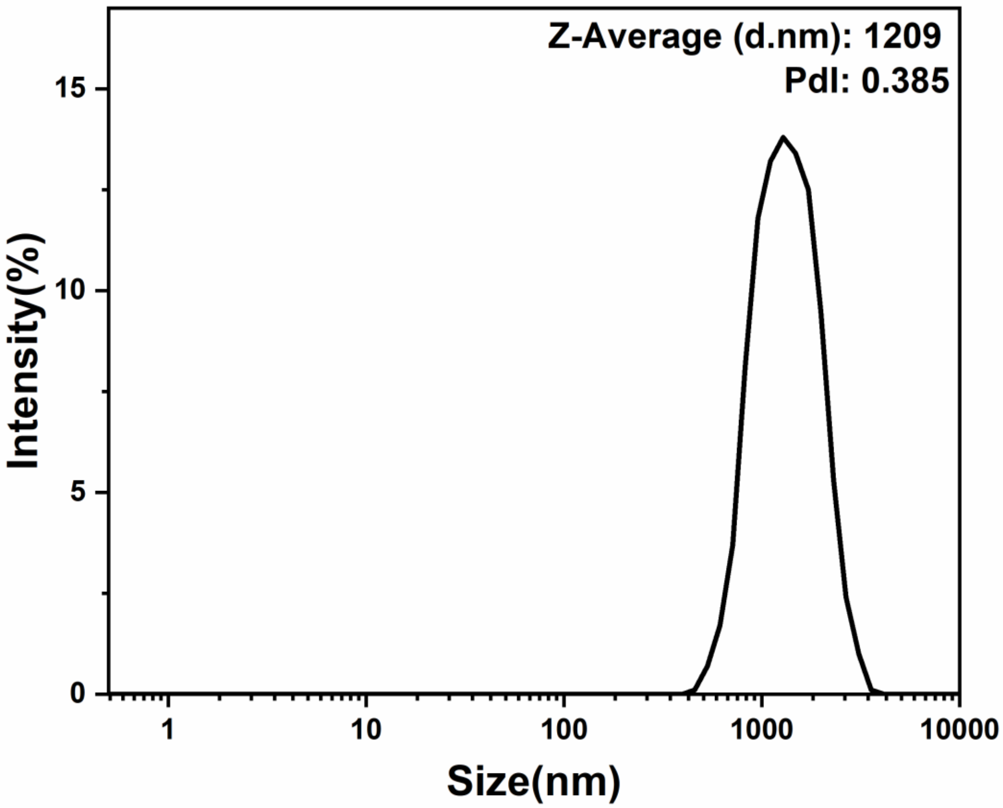

Fig. S4 Dynamic light scattering (DLS) distributions of silver nanodimers with a post-ultrasound washing step for 2 h (increased size due to particle aggregation).

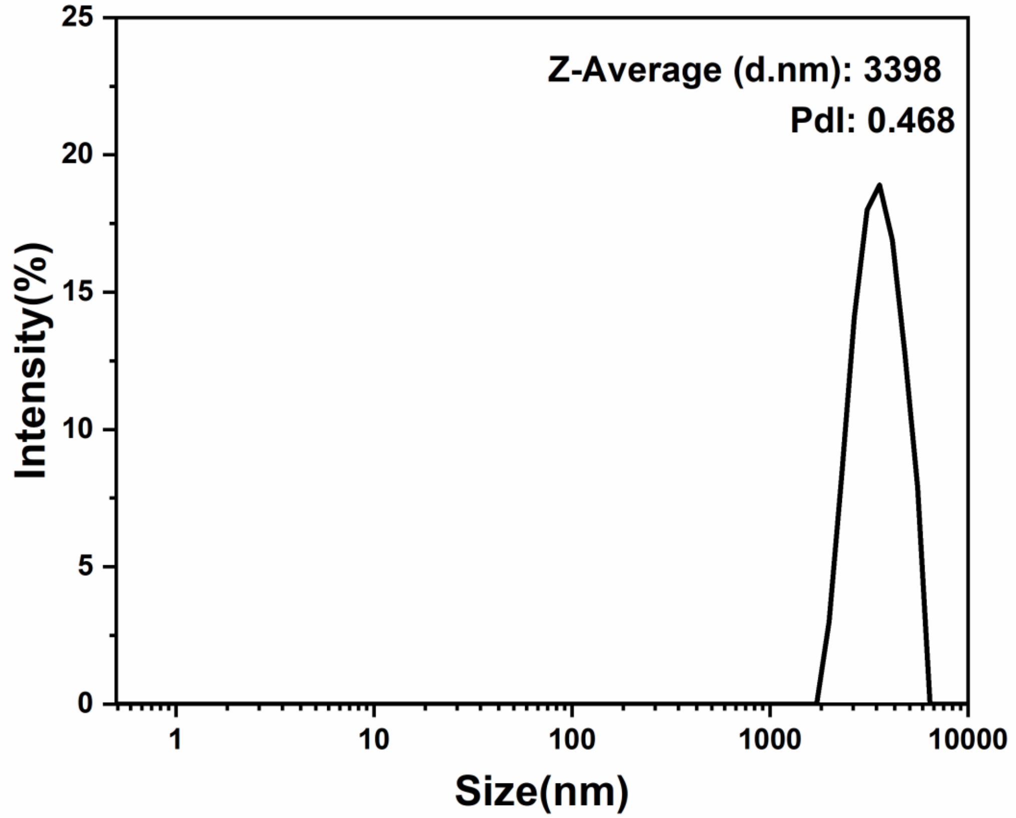

Fig. S5 Dynamic light scattering (DLS) distributions of silver nanodimers with a post-ultrasound washing step for 5 h (increased size due to particle aggregation).

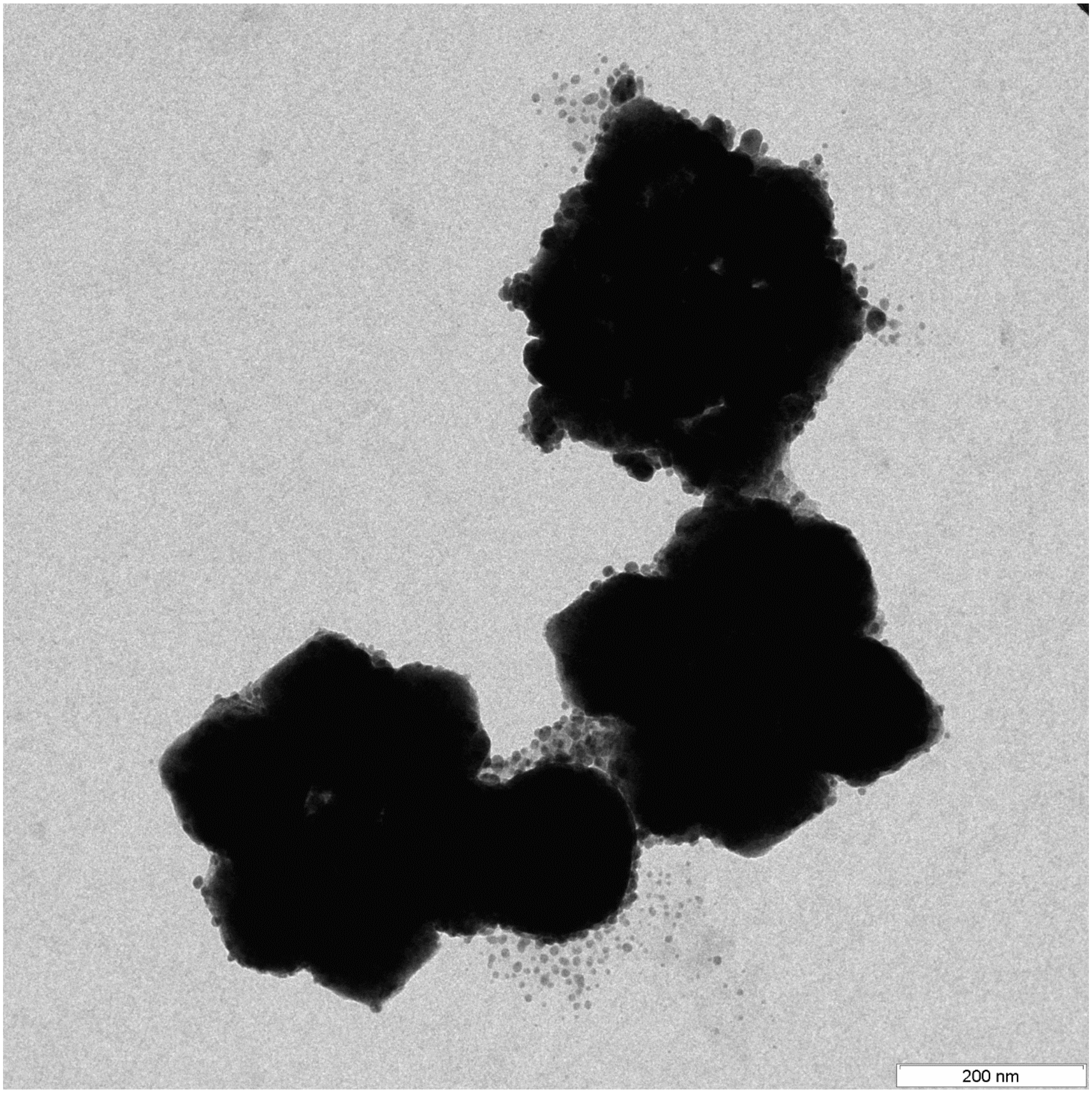

Fig. S6 TEM image of nanodimers obtained by polyol process with pre-sonication for 30 min.

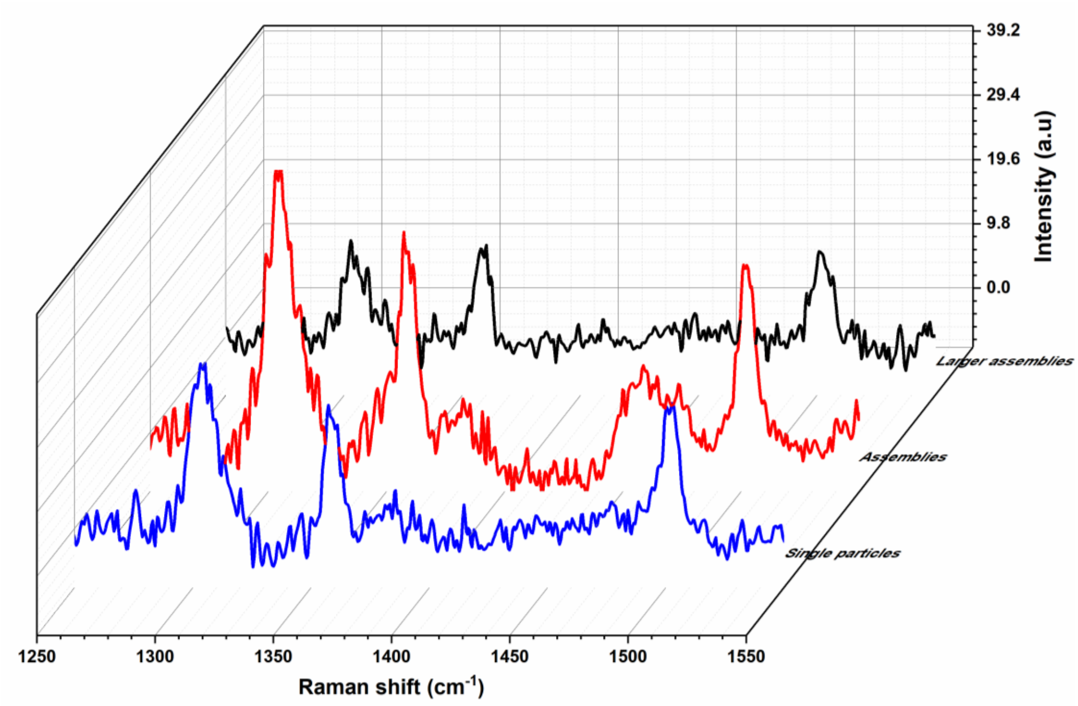

Fig. S7 SERS spectra of different assemblies (blue for single particles, red for assemblies corresponding to the characterization reported in Fig. S4, black for larger assemblies corresponding to the characterization reported in Fig. S5) with $1x10^{-6}$ M Rhodamine 6G (R6G) in aqueous solution. The experiments were performed with a Raman microscope (DXR, Thermo Fisher) with a 780 nm frequency-stabilized single mode diode laser.

Table S1 Content of different elements on nanodimers analysed by EDX.

Element	C	O	Na	Si	Ag	Total
Spectrum 1	5.20	9.99	1.16	3.01	80.63	100.00
Spectrum 2	3.88	5.28	0.39	1.70	88.75	100.00
Spectrum 3	3.64	2.30	0.98	0.38	92.70	100.00
